# Aging in high functioning elderly persons: study design and analyses of behavioral and psychological factors

**DOI:** 10.1111/sms.13368

**Published:** 2019-04-29

**Authors:** Thomas Finkenzeller, Birgit Pötzelsberger, Alexander Kösters, Sabine Würth, Günter Amesberger, Flemming Dela, Erich Müller

**Affiliations:** ^1^ Department of Sport Science and Kinesiology University of Salzburg Salzburg Austria; ^2^ Xlab Center for Healthy Aging Department of Biomedical Sciences University of Copenhagen Copenhagen Denmark; ^3^ Department of Geriatrics Bispebjeg University Hospital Copenhagen Denmark

**Keywords:** active lifestyle, longitudinal study, multidisciplinary research, protective resources, successful aging

## Abstract

This article aims to (a) describe the study design of a 6‐year follow‐up multidisciplinary research project on aging, (b) report the psychosocial characteristics of the sample in detail, and (c) evaluate aging‐related changes of health, physical activity, and psychosocial characteristics in 10 young‐old (age at pre‐test: *M* ± *SD* = 63.2 ± 1.5) and 12 old‐old (age at pre‐test: *M* ± *SD* = 69 ± 2) individuals. Both age groups consist of individuals displaying a high health status, a high extent of physical activity, high levels of psychosocial properties in the dimensions of well‐being, life satisfaction, self‐concept, body image, self‐esteem, and self‐efficacy, as well as a low general depression index. Psychosocial characteristics demonstrated a stable pattern over a period of nearly 6 years in both age groups with the exceptions of physical activity, satisfaction with children, general depression, and self‐efficacy. Furthermore, physical self‐concept decreased in old‐old adults, whereas the young‐olds showed no change. We assume that a high psychosocial status and a physically active lifestyle play an important role for mastering aging successfully in two life phases, each of which has its own challenges for older individuals. The decline in the physical self‐concept of old‐olds is interpreted as a first sign of subjective aging. Its association with losses in physical performance should be addressed in future studies. Finally, aging‐related changes should be monitored on an individual level in order to capture the complex dynamic of aging that is not considered in analyses of between‐person differences or averages.

## 1 INTRODUCTION

It is well established that healthy aging goes along with a more gradual decline in physical^1^, ^2^ and cognitive functioning.^3^, ^4^, ^5^ Strong empirical evidence supports the protective effect of an active lifestyle resulting in decelerated age‐related degeneration processes.^6^, ^7^, ^8^ Even so, there is a debate about when decline begins and how high levels of psychosocial characteristics^9^ influence age‐related changes. The focused psychosocial factors within this study refer to psychological and social factors which include individual‐level processes and meanings that influence mental states as well as general factors at the level of human society concerned with social structure and social processes that impinge on the individual.^10^

Transition from late midlife (<65 years) to late life (≥65 years)^11^ is a critical phase that is accompanied by numerous processes and adjustments on the biological, societal, interpersonal, and psychological domains.^12^ A transitional period, however, represents a developmental task of terminating an existing structure and working toward the initiation of a new structure.^13^ In particular, individuals who are in the period of late midlife to late life are faced with various challenges such as the transition to retirement or the transition to retirement of the partner, coping with more time resources in general, as well as coping with more time with the partner, less social contact with former colleagues in addition to other lifestyle changes. Numerous studies have shown that the transition from late midlife to late life is a period of change on multiple domains such as physical activity,^14^ well‐being,^15^ social network,^16^ life satisfaction,^17^ self‐esteem, and depression.^18^ Kim and Moen^19^ point out that in this critical life phase, economic, personal, and social‐relational resources may positively affect psychological well‐being, and therefore, may encourage its decelerated age‐related decline.

Baltes and Lang^20^ were among the first researchers to examine psychological and social factors on aging with a special focus on successful agers. They emphasized the protective role of psychological and social resources for adaptive processes. According to the MacArthur model,^21^ successful aging refers to low probability of disease and disease‐related disability, high cognitive and physical functional capacity, and active engagement with life. Depp and Jeste^22^ identified the following as components of successful aging: disability/physical function, cognitive functioning, life satisfaction/well‐being, social/productive engagement, absence of illness, longevity, environment/finances, and self‐rated successful aging. Their review underlines the importance of psychosocial factors for aging. A further study by Rowe and Kahn^23^ supports the notion that social factors have an important impact on the capacity of individuals to age successfully. In a comprehensive multivariate research, Chou and Chi^24^ defined successful aging by functional, affective, and cognitive status, and productive involvement. Using a multiple regression model for the prediction of successful aging, they found that the factors self‐rated health, number of close relatives, frequency of contact with friends, years of education, and life satisfaction correlated significantly with successful aging in a positive way. Age, financial strain, number of chronic illnesses, and hearing impairment demonstrated significant negative relationships. Furthermore, being of female gender had a positive impact on successful aging.

In summary, few studies^25^, ^26^, ^27^, ^28^ have examined successful aging under a comprehensive psychosocial perspective considering psychological constructs like well‐being, life satisfaction, self‐efficacy, self‐concept, and subjective health. There are strong indices that high levels in self‐efficacy and resilience^29^ positively influence quality of life in older adults.

To extend knowledge on successful aging, Pruchno et al^30^ proposed to assess subjective ratings and objective values, for instance of cognitive and physical performance. Thus, this project aims to record objective and subjective data, including the aforementioned components of Depp and Jeste.^22^ Furthermore, physiological and physical parameters are analyzed to assess aging‐related decline in a holistic manner.

The multidimensional project at hand addresses how aging‐related changes proceed in two age groups of elderly persons exhibiting high psychological and physiological functioning baseline levels. Both age groups have to face critical life circumstances as transition to retirement or an advancing aging process, both of which require particular coping mechanisms. High subjective ratings of psychosocial competencies are seen as resources that may facilitate the mastering of these life phases with small losses in the psychosocial, cognitive, and/or physiological domains.

The aims of this article are threefold. First, the study design and the comprehensive test battery are presented. Second, data on physical activity and psychosocial characteristics are reported with respect to comparative values aiming at a deeper understanding of the subjects’ psychological traits and state levels that will support the anchoring and interpretation of the results addressed in other articles on this research topic. Third, long‐term changes in physical activity and psychosocial characteristics of two age groups of elderly persons of whom are highly functional, both mentally and physically, are analyzed. In a first step, young‐old persons who are at the age of retirement are compared to old‐old persons who have already reached the post‐retirement phase. In a second step, a follow‐up assessment with a 6‐year interval should add new knowledge about the stability of physical activity and psychosocial variables in the two age groups of elderly persons.

## 2 METHODS

### 2.1 Subjects

The subjects were recruited from a sample of a previous study, the Salzburg Skiing for the Elderly Study (SASES).^31^ The SASES sample is characterized by above average values in psychological well‐being and psychosocial variables like life satisfaction, self‐concept, health status, and self‐efficacy.^32^ Thus, we conclude that the sample is appropriate to test long‐term changes of elderly persons who are highly mentally and physically. The local ethics committee approved this study and participants gave their written informed consent. All subjects who participated at the retention test of the SASES (named as t1 in this article) were invited to perform tests that were administered in the SASES in a follow‐up measurement. In total, 25 subjects out of 42 potential candidates agreed to take part again. Out of these, three subjects were excluded due to stroke (n* *=* *1), heart disease (n* *=* *1), and vacation at t1 (n* *=* *1). Reasons for nonparticipation in the follow‐up were manifold ranging from not being able to participate at the fixed measurement time due to vacation or other reasons (n = 3), no motivation to participate again (n* *=* *7), lost to follow‐up (n* *=* *6), and injury (n* *=* *1).

Hence, the sample of this study is the result of a diverse selection process. Information on age, weeks of sickness and educational status is included in Table 1. According to their ages, seven females and three males were assigned by age to the young‐old (<66 years), and six females and six males to the old‐old group (≥66 years) which comes close to the classification of Xu, Qiu, Gatz, Pedersen, Johansson, Fratiglioni.^11^ Thirteen subjects took (n_young‐old_ = 8, n_old‐old_ = 5) drugs regularly for medical reasons. However, only six subjects suffered from chronic diseases such as coronary heart disease (n_young‐old_ = 1, n_old‐old_ = 1), diabetes mellitus (n_young‐old_ = 2), hypertension (n_old‐old_ = 1), sleeping disorders (n_young‐old_ = 1), prostate cancer (n_old‐old_ = 1), or hypercholesterolemia (n_young‐old_ = 1). In two articles within this supplement, the sample was divided in another way corresponding to the initial assignment of the SASES study to an alpine skiing intervention (IG) and a control group (CG). In the group of young‐olds are five persons each of the IG and CG, and in the group of old‐olds six persons each of the IG and CG.

**Table 1:** Age, weeks of sickness and educational status. Data are shown as mean ± SD

	Young‐old (n* *=* *10)	Old‐old (n* *=* *12)	All (n* *=* *22)
Age (years)			
2009 (t1)	63.2 ± 1.5	69.0 ± 2.0	66.4 ± 3.4
2015 (t2)	69.1 ± 1.5	74.8 ± 2.3	72.2 ± 3.5
Weeks of sickness per year			
2010 (n* *=* *2 young‐old; n* *=* *4 old‐old)	0.3 ± 0.7	0.6 ± 0.9	0.5 ± 0.8
2011 (n* *=* *2 young‐old; n* *=* *3 old‐old)	0.5 ± 1.1	0.4 ± 0.8	0.5 ± 0.9
2012 (n* *=* *2 young‐old; n* *=* *3 old‐old)	0.3 ± 0.7	0.4 ± 0.8	0.4 ± 0.7
2013 (n* *=* *2 young‐old; n* *=* *5 old‐old)	0.4 ± 1.0	1.6 ± 2.7	1.1 ± 2.2
2014 (n* *=* *5 young‐old; n* *=* *5 old‐old)	1.7 ± 2.2	1.4 ± 2.6	1.6 ± 2.4
Education (frequencies)			
Compulsory school (9‐10 y)	1	4	5
Vocational school (10‐12 y)	7	4	11
Qual. for university entrance (12‐13 y)	0	4	4
University degree	2	0	2

### 2.2 Study design/Procedure

**Figure 1:**
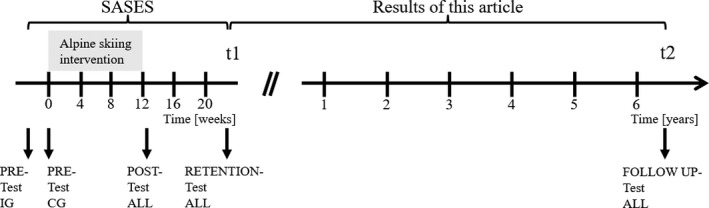
Study design of the Salzburg Skiing for the Elderly Study extended by a follow‐up measurement. Pre‐test intervention group (IG): December 9‐20, 2008; control group (CG): January 7‐15, 2009. Intervention Phase: January 7‐March 28, 2009. Post‐test IG + CG: March 30‐April 6, 2009. Retention‐test ALL (t1): June 2‐July 1, 2009. Follow‐up test ALL (t2): April 20‐25, 2015

The test battery of the SASES^31^ was conducted one more time 5 years and 10 months after the retention test of SASES in a slightly shortened version and marginally changed ordering of the tests within the test battery (Table 2). The first three measurements of SASES were conducted with a period of 12 months between pre‐ and post‐test, and a retention test 2 months later. The results of the SASES evaluation of the impact of a guided alpine skiing intervention were published previously.^33^ Regarding the research question, analyses of this article exclusively include data of the retention test (t1) and data of the follow‐up test (t2). Other articles within this supplement consider all measurements to answer their research questions. An overview of the overall study design and measurements is provided in Figure 1.

**Table 2:** Test battery applied at t1 and t2, including information on measurement day and ordering of the tests within a test day

Test day		Order		Test	Content/Description
t1	t2	t1	t2		
1st	1st	1	2	Anthropometric parameters	Body weight, body height, body composition
1st	1st	2	3	Muscle morphometry	Ultrasound measurement
1st	1st	4	5	Strength endurance	Strength endurance (leg press)
2nd	2nd	1	2	Muscle strength	Isometric maximum strength (leg extension/flexion)
2nd	1st or 2nd	2	6 or 1	Psychological assessments	Questionnaires, cognitive performance, psychophysiological data
3rd	1st	1	1	Blood sample	Cardiovascular risk profile
3rd	2nd	2	3	Endurance	Cardiorespiratory fitness (Bicycle ergometer)

### 2.3 Questionnaires

The same comprehensive test battery of questionnaires as was applied in SASES was administered at t2, with the exception of the dimensions of profession (no one held down a full‐time job) and marriage/partnership (in order to avoid unpleasant emotions aroused by eg, death of the partner). Scientifically well‐established and evidently appropriate questionnaires for successful aging^22^ assessing aspects of behavioral, psychological and social factors were administered. The test battery includes rating scales of physical activity, well‐being (state), life satisfaction (including social factors), physical self‐concept, body image, health state, general depression, general self‐concept, and self‐efficacy. Table 3 presents the entire test battery including information on scaling and Cronbach's *α*. Information on reference values is given in Table 4.

**Table 3:** Description of applied questionnaires

Questionnaire	Abbrev.	Dimensions	Likert scale	Cronbach's *α*
Physical activity^34^	PA	Exercise time per week	5/4 steps	n.a.
Multidimensional well‐being scale^41^	MDBF‐A	Good/bad mood, alertness/fatigue, calmness/agitation	5 steps	0.76‐0.94
Questionnaire on life satisfaction^42^	FLZ	Health, profession^a^, financial status, marriage/partnership^a^, relationship to children, own person, friends and relatives, accommodation	7 steps	0.84‐0.95
Physical self‐concept scale^34^, ^43^	PSK	Flexibility, coordination, strength, speed, endurance, general sportiness	4 steps	0.84‐0.90
Body image scale^44^	KSB‐short	Positive attitude toward the body, worries about figure, perceived fitness state	4 steps	0.69‐0.73
Health^45^	GES	Actual health state, general health state	5 steps	0.85
General depression scale^46^	ADS‐K	General depression	4 steps	0.90
The Frankfurter self‐concept scales^47^	FSKN	Social self‐esteem, Performance related self‐esteem	6 steps	0.70‐0.86
Self‐efficacy^48^	SW	General self‐efficacy	4 steps	0.91

*Note.*

a Not applied at t2.

**Table 4:** Descriptive statistics on physical activity and psychological characteristics for the samples of young‐olds and old‐olds including reference values. Data are mean ± SD

Quest.	Dimension	Young‐old n t1/t2	t1	t2	Old‐old n t1/t2	t1	t2	Ref. value (Table 3)
PA	Phys. activity (min/wk)	10/10	579.5 ± 237.7	527.5 ± 286.7	12/12	637.1 ± 331.3	475.8 ± 306.8	
MDBF‐A	Good/bad mood	9/9	17.1 ± 2.5	16.9 ± 2.7	12/12	17.3 ± 1.6	16.5 ± 2.5	15.0 ± 3.5
	Alertness/fatigue	9/9	16.0 ± 3.2	14.9 ± 3.1	12/12	16.8 ± 2.6	15.6 ± 1.8	12.7 ± 4.0
	Calmness/agitation	9/9	15.9 ± 3.7	15.9 ± 2.4	12/12	16.8 ± 3.0	15.4 ± 3.0	14.0 ± 3.4
FLZ	Health	10/10	41.8 ± 3.6	40.1 ± 4.6	12/12	42.0 ± 4.3	41.1 ± 4.4	34.8 ± 7.6
	Profession	10/10	41.2 ± 5.7	Not asked	12/12	39.3 ± 8.9	Not asked	Rephrased
	Financial status	9/9	39.2 ± 4.9	36.1 ± 7.8	11/11	39.5 ± 7.2	40.2 ± 5.7	35.3 ± 7.6
	Marriage/partnersh.	10/10	42.1 ± 5.2	Not asked	11/11	37.4 ± 7.9	Not asked	38.7 ± 8.0
	Relationsh. w. children	7/7	45.0 ± 4.6	40.6 ± 1.5	12/12	40.7 ± 5.6	37.2 ± 3.7	38.7 ± 6.6
	Own person	10/10	41.9 ± 4.5	41.9 ± 4.5	12/12	40.8 ± 3.9	41.5 ± 1.5	38.2 ± 5.7
	Friends and relatives	10/10	40.9 ± 4.2	42.9 ± 3.4	12/12	36.7 ± 7.3	38.8 ± 4.7	37.3 ± 6.1
	Accommodation	10/10	44.4 ± 3.6	44.8 ± 2.9	12/12	43.1 ± 3.2	43.9 ± 4.2	39.1 ± 6.1
SSL	Flexibility	10/10	3.2 ± 0.6	3.3 ± 0.7	12/12	3.4 ± 0.5	3.2 ± 0.5	2.9 ± 0.7
	Coordination	10/10	2.9 ± 0.7	2.9 ± 0.6	11/11	3.3 ± 0.3	3.0 ± 0.3	2.8 ± 0.6
	Strength	10/10	2.8 ± 0.6	2.7 ± 0.8	10/10	3.2 ± 0.3	3.0 ± 0.4	2.4 ± 0.7
	Speed	10/10	2.7 ± 0.8	2.5 ± 0.7	11/11	3.3 ± 0.3	2.7 ± 0.5	2.4 ± 0.7
	Endurance	8/8	1.9 ± 0.8	1.9 ± 0.7	12/12	2.6 ± 0.8	2.2 ± 0.5	2.1 ± 0.7
	General sportiness	7/7	2.6 ± 0.9	2.7 ± 0.8	11/11	3.0 ± 0.6	2.5 ± 0.5	2.3 ± 0.6
KSB	Pos. attit. tow. body	8/8	3.2 ± 0.5	3.3 ± 0.3	11/11	3.2 ± 0.5	3.1 ± 0.5	2.9 ± 0.6
	Worries about figure	8/8	2.4 ± 1.0	2.5 ± 1.1	12/12	1.9 ± 0.5	1.9 ± 0.6	1.7 ± 0.7
	Perc. fitness state	10/10	3.2 ± 0.5	2.7 ± 0.7	12/12	3.3 ± 0.5	2.9 ± 0.7	2.8 ± 0.8
GES	Actual health state	10/10	4.2 ± 0.4	4.0 ± 0.7	12/12	4.2 ± 0.6	4.1 ± 0.8	3.7 ± 0.7
	General health state	10/10	4.1 ± 0.6	3.8 ± 0.6	12/12	4.33 ± 0.7	4.3 ± 0.5	
ADS‐K	General depression	9/9	7.3 ± 5.6	9.3 ± 5.7	11/11	6.7 ± 3.9	7.6 ± 5.1	10.3 ± 8.5
FSKN	Social self‐esteem	10/10	4.7 ± 0.6	4.9 ± 0.7	12/12	5.2 ± 0.7	5.0 ± 0.5	4.7 ± 0.7
	Perf. rel. self‐esteem	10/10	4.5 ± 0.6	4.7 ± 0.4	12/12	4.9 ± 0.5	4.8 ± 0.3	4.8 ± 0.6
SW	General self‐efficacy	10/10	3.1 ± 0.4	3.0 ± 0.4	12/12	3.3 ± 0.5	3.1 ± 0.5	3.1 ± 0.5

*Note.*

minutes per week (min/wk).

In contrast to SASES, all questions of the follow‐up test were asked relating to the time span of the last 5 years (“When answering, please reminisce about the last five years.”). The questionnaires were administered as paper‐pencil‐tests.

At t1 and t2, the average duration and frequency per week of long walks for leisure‐time or for an errand, bicycling, gardening and indoor work, as well as other activities (free answer possibility) were assessed in accordance with Stiller.^34^ The total exercise time per week was computed by multiplying the duration and the frequency of each activity. Subsequently, the values were totaled. Test‐retest reliability of total exercise time using product‐moment correlation between t1 and t2 is at *r*(20) = 0.75, *P *= <0.001. At t2, weeks of sickness per year (2010‐2014) were recorded retrospectively (Table 1).

### 2.4 Statistics

All statistical analyses were performed using the software IBM SPSS Statistics for Windows (Version 23.0; IBM Corp., Armonk, NY, USA). The data of t1 and t2 are presented as mean (*M*) and standard deviation (*SD*). Most of the variables demonstrated normal distribution according to the Shapiro‐Wilk test. The few nonnormal distributed variables violated the assumption of normality marginally, thus, only parametric tests were used in accordance with Nimon.^35^ Alterations in weeks of sickness per year were analyzed by using a 2 × 2 × 5 (Sex [male, female] × Age Group [young‐old, old‐old] × Time [five measurements]) multivariate analyses of variances with repeated measures (MANOVA‐RM). For the other constructs, 2 × 2 × 2 (Sex [male, female] × Age Group [young‐old, old‐old] × Time [pre‐test, post‐test]) MANOVA‐RM were performed with the factors sex, age group, and time to assess aging‐dependent effects. In the case of significant main effects, follow‐up univariate tests for each variable of the construct using the same factors as in the MANOVA were calculated. The significance level was set at *P *<* *0.05.

## 3 RESULTS

Descriptive statistics on questionnaire results of t1 and t2 are depicted in Table 4, including reference values of the literature that are cited in Table 3. Comparisons to normative data and data from similar samples, respectively, revealed above average values in the dimension health for the questionnaire on life satisfaction, and in the single items on general and actual health at both measurements. The subjects of the young‐old group showed a slightly reduced average value in the dimension for endurance of self‐concept by the sports performance scale at both measurements (−9.52% at t1 + t2), and in the perceived fitness state of the body image scale (−3.57) at t1 as the reference group. Furthermore, young‐olds demonstrated lower average scores in performance‐related self‐esteem at both measurements (t1: −6.25%; t2: −2.08%) as well as in general self‐efficacy at t2 (−3.22%) in comparison with the reference group. Slightly lower average values compared with reference values were observed for subjects of the old‐old group in the life satisfaction dimensions of marriage/partnership (−3.36%) and friends and relatives (−1.61%) at t1, as well as for relationship with children (−3.88%) at t2. The general depression average score was lower for both groups than the reference value at t1 and t2. Three subjects demonstrated an above average depression score at t1 and seven subjects at t2.

Questionnaire results of (M‐)ANOVA‐RM for the factor time, for the factor age group and the interaction of time and age group are shown in Table 5. Significant decreases over time were obtained for the univariate constructs of physical activity and general self‐efficacy. The general depression score increased significantly from t1 to t2. A global significant decrease over time was apparent for life satisfaction. Post hoc analyses demonstrated that this effect is related to the dimension satisfaction with the relationship with children, *F*(1, 13) = 12.86, *P *<* *0.01, *η* ^2^ = 0.50.

**Table 5:** Results of 2 × 2 × 2 (M)ANOVA‐RM on each assessed construct

Construct	Quest.	Young‐old n	Old‐old n	Time effect				Age group effect				Time × Age group			
				*df*	*F*	*η* ^2^	*P*	*df*	*F*	*η* ^2^	*P*	*df*	*F*	*η* ^2^	*P*
Sickness wk/y (2 × 2 × 5 ANOVA‐RM)	Single item	10	12	4, 72	3.16	0.15	0.08	1, 18	0.05	0.01	0.82	4, 72	0.89	0.05	0.38
Physical activity	PA	10	12	1, 18	7.62	0.30	0.01*	1, 18	0.15	0.01	0.70	1, 18	0.59	0.03	0.45
Well‐being	MDBF‐A	9	12	3, 15	1.24	0.20	0.33	3, 15	1.14	0.19	0.36	3, 15	0.84	0.14	0.49
Life satisfaction	FLZ	6	11	6, 8	8.37	0.86	<0.01**	6, 8	4.60	0.78	0.03*	6, 8	0.63	0.32	0.70
Self‐concept of sports performance	SSL	6	10	6, 7	0.92	0.44	0.53	6, 7	1.90	0.62	0.21	6, 7	6.53	0.85	<0.05*
Body image	KSB	7	11	3, 12	3.37	0.46	0.06	3, 12	0.94	0.19	0.45	3, 12	0.11	0.03	0.95
Actual and general health	GES	10	12	2, 17	1.07	0.11	0.37	2, 17	3.39	0.29	0.06	2, 17	0.37	0.04	0.70
General depression	ADS‐K	9	11	1, 16	5.77	0.27	0.03*	1, 16	0.69	0.04	0.42	1, 16	1.37	0.08	0.26
Self‐esteem	FSKN	10	12	2, 17	0.45	0.01	0.96	2, 17	1.23	0.13	0.32	2, 17	1.22	0.13	0.32
General self‐efficacy	SW	10	12	1, 18	5.23	0.23	0.04*	1, 18	1.06	0.06	0.32	1, 18	0.04	0.01	0.84

*Note.*

weeks per year (wk/y); *P <* *0.05; **P <* *0.01.

The factor age group exhibits a significant overall effect in life satisfaction. However, post hoc analyses revealed no significance on a univariate level.

The multidimensional self‐concept of sports performance shows a significant interaction of time and age group. Post hoc analyses indicated significant interactions in the dimensions of coordination [*F*(1, 12) = 13.07, *P *<* *0.01, *η* ^2^ = 0.52), speed [*F*(1, 12) = 5.25, *P *<* *0.04, *η* ^2^ = 0.30], and general sportiness [*F*(1, 12) = 10.22, *P *<* *0.01, *η* ^2^ = 0.46]. The sample of young‐olds remained stable, whereas the old‐olds assess their coordination, speed, and general sportiness lower at t2.

There are no significant results on the factor sex, the interaction of time and sex, and the interaction of sex × age group × time. A significant interaction of sex and age group was obtained in life satisfaction, *F*(6, 8) = 3.86, *P *=* *0.04, *η* ^2^ = 0.74. Post hoc analyses indicate that the interaction is present in the dimension satisfaction with oneself (scale “own person”), *F* (1, 13) = 10.51, *P *=* *0.01, *η* ^2^ = 0.45. Male persons of the young‐olds sample had lower values than females in this age group, whereas in the old‐old group the trend is in the opposite direction.

## 4 DISCUSSION

This article reports the overall study design of multidisciplinary research on aging‐related changes in two different age groups of elderly persons who have a high number of protective resources available. A special feature of this research is the recruitment of subjects from a former study, and the continuation of that same test battery in this study.

The psychosocial characteristics of this sample at t1 were similar to the previously reported baseline values of the SASES.^32^ In general, the sample of this study offers the possibility to evaluate aging‐related declines in the biological, physical and cognitive domain of subjectively healthy, rarely sick and very active individuals that possess predominantly above average psychosocial characteristics in the dimensions of well‐being, life satisfaction, self‐concept of sports performance, body image, general depression (lower than average), self‐esteem, and general self‐efficacy. Hence, the sample is suited to extend knowledge on aging‐related alterations in resource‐rich persons in the psychosocial domain considering age by dividing the sample into two age groups.

Despite the fact that there was a strong variability of the subjects’ medical histories and diseases, both age groups demonstrated above average psychosocial characteristics in nearly all scales compared to norm data and data from similar studies, respectively, at t1 indicating that the recruitment of successful agers in the psychosocial domain succeeded. Even on the individual level, personal subjective assessments of health in the subscale of life satisfaction, as well as in the single items on health were above average values of the reference group in all subjects. With regard to the life satisfaction scales, only four subjects answered below the reference average in two or three of the remaining six subscales supporting the assumption of having subjects who are very satisfied with their lives.

A more differentiated picture can be drawn of body image, self‐concept of sport performance, self‐esteem, and self‐efficacy. A higher number of individual assessments below reference and comparative average values are recorded at both measurements. There is an inconsistent distribution of these below average individual values on the different constructs reflecting its complex interdependency.

### 4.1 No differences in age groups

Although the two age groups differed 5.65 years in average age, which comes close to the time interval between t1 and t2, the two groups demonstrated no differences in weeks of sickness, in physical activity or in psychosocial characteristics. When planning the study, the expectation was that young‐olds would show at t2 similar results as the old‐olds at t1. The sampling failed to confirm this expectation, which hindered the ability to draw conclusions that are more precise concerning the impact of aging and crucial life phases.

### 4.2 Time effects

With the exception of one subject (85 minutes at t1 and 150 minutes at t2 of physical activity per week), all subjects rated their physical activity above 150 minutes per week at t1 and t2 which is above the recommendation of the American College of Sports Medicine (ACSM).^36^ However, there were extreme individual differences ranging from 150 to 1260 minutes of physical activity per week at t2. The decrease found in physical activity after the age of 65 years is well in line with Boisvert et al,^37^ whereas Barnett et al^14^ provide evidence for an increase in physical activity after the retirement transition. This inconsistency is indicated on the individual level by the results of this study. Despite the significant decrease in physical activity, seven subjects increased their physical activity by as much as 20‐290 min/wk, arguing for evaluating individual differences of age‐related changes in physiological and physical aspects. Why some subjects increased, and others decreased physical activity during a period of nearly 6 years can have many reasons and cannot be concluded by the data of this study. Similarly, critical life events and illnesses delivered no reasonable explanation for changing physical activity. The subjective rating of physical activity may also have influenced the results on the change of physical activity.

There were no differences in weeks of sickness from 2010 to 2014, supporting the stability of a high general and actual health state. These results should be considered with caution because questions with regard to weeks of sickness per year were asked retrospectively in the year of 2015. One might remember weeks of illness better when experienced more recently; therefore, the aging‐related effects of illness might be estimated differently regarding the time span. Nevertheless, the number of weeks of illness is low with 1.55 weeks per year at the end of the study.

Findings on psychosocial variables demonstrated stability over a time span of nearly 6 years in both age groups with the exceptions of satisfaction with children, general depression and self‐efficacy. This is remarkable because the values in these variables at t1 demonstrated high levels and it is likely that with increasing age, constructs such as well‐being, life satisfaction,^38^ self‐concept, and self‐esteem^39^ will decline. The significant decrease in satisfaction with children and general depression was still at reference average level at t2. The only slightly reduced average value was observed at t2 concerning self‐efficacy in comparison with norm data. Concerning psychosocial variables, the conclusion is that the young‐olds, who are challenged with the transition into retirement, and the old‐olds, who may be faced with more ongoing aging processes, both successfully mastered the time span of nearly 6 years between research projects. According to Pinquart and Schindler,^17^ it is assumed that the high level of psychosocial characteristics represents a resource that may have facilitated the maintenance of the above average level in nearly all psychosocial domains. This assumption is well in line with Chou and Chi,^24^ who emphasized the importance of close relatives, contact with friends, financial security, absence of chronic illness, high self‐rated health, high life satisfaction, and further aspects for successful aging. Changes in general depression and self‐efficacy might be initial predictors of a slight aging‐related decrease that might be connected to a loss of physical and cognitive performance. Longitudinal studies are warranted to evaluate the interdependence of psychosocial variables in order to extend knowledge on aging‐related psychological dynamics.

There were strong individual differences in aging‐related changes from t1 to t2 in the depression score, satisfaction with children, and self‐efficacy. Initially, the approach of this project was based on between‐person differences and averages without regarding individual specificities in psychosocial variables. However, results on psychosocial variables underline the assumption that aging is highly individualized,^40^ and therefore, individual changes in physical activity and psychosocial characteristics will be considered in further articles of this project.

### 4.3 Interaction of age group and time (aging)

Aging‐dependent changes seem to interact with age in the self‐concept of sport performance. The subjective assessment of coordination, speed, and general sportiness demonstrated a decrease in the old‐olds, whereas young‐olds stayed quite stable. This result delivers the first fruitful information on aging‐related changes in the physical concept that should be pursued in future studies as already recommended by Amesberger et al.^33^ An important aspect on which this project focused is how the decrease in the physical self‐concept is interrelated to changes in objective physical performance (reported within this issue).

In summary, both age groups of this study consist of individuals possessing high levels of psychosocial characteristics and physical activity that are quite stable, even after a time interval of nearly 6 years. Hence, both age groups are appropriate for demonstrating aging‐related changes in the biological, physical, and cognitive domains of subjects who are resource rich in the psychosocial domain.

### 4.4 Limitations

Conclusions should be drawn with caution considering the high dropout rate. At t1, the participants who dropped out were not significantly different from the participants who finished the study. Unfortunately, no knowledge exists about possible alterations between t1 and t2, which could have led to the withdrawals. This has to be taken into account when looking at the above average psychosocial characteristics of the analyzed sample. Furthermore, the low sample size is associated with low statistical power reducing the likelihood of determining significant effects. The assessment of physical activity through questionnaire has to be regarded as a limitation. Moreover, a comparison group including subjects having lower physical activity and psychosocial scores was not implemented because of the genesis of the study design. One may only speculate how aging‐related changes will occur in individuals that do not benefit from numerous resources in the psychosocial domain. In addition, contextual information on changes in life circumstances was not recorded between the period of late midlife and late life. Finally, aging‐related changes were evaluated by only two measurements. Nonetheless, the strengths of the study represent the longitudinal design, multidisciplinary perspective, the familiarity with the test battery, and the focus on elderly persons having a high number of protective resources available to them.

## 5 PERSPECTIVE

This study showed high and stable perceptions of the psychosocial state and trait components over a time course of nearly 6 years in elderly individuals. We assume that high psychosocial competencies represent a protective resource against a decline in these concepts. This assumption needs to be tested in future studies using higher sample sizes and more sophisticated research designs considering individual courses. Thus, in order to facilitate successful aging, promotion of the development of strong and numerous psychological and social competencies is recommended. Furthermore, decreases in the physical self‐concept were observed only in the old‐old adults, possibly reflecting an ongoing associated decrease in objective physical performance. The relationship of the physical self‐concept and objective performance, as well as results in the cognitive, physical and physiological domain are reported in further articles within this supplement in order to extend knowledge on the protective value of high physical activity and psychosocial components.
